# Close of the Volume

**Published:** 1859-05

**Authors:** 


### EDITORIAL DEPARTMENT.

*Close of the Volume.*—This number closes our fourteenth volume, and finishes the first year of our sole editorial responsibility; we take advantage of the little space which a press of original matter has left us to acknowledge the kindness of our contributors and subscribers who have consented to continueto us the favors which they extended to the former editors. Relieved, as we now are, of the business matters of the Journal, a department for which we had but little taste, and confident that its temporal condition is good and improving, we will commence upon Vol. XV with a light heart, hoping that our growing experience may make our editorial capabilities more commensurate with its requirements.

A large portion of this number is devoted to a review of the Transactions of the American Medical Association; and our readers will readily see why it could not have been deferred till another issue. This, however, excludes eclectic matter, and permits only to say a word in our editorial department; we have, however, done some labor for this number, in the preparation of our index, which we have made unusually comprehensive. Several contributions, letters, and a description of Palmer's artificial leg and arm with uts, stand over for our next.

